# Dermoscopic Features of Foreign Body Cutaneous Granuloma: A Case Series

**DOI:** 10.5826/dpc.1103a25

**Published:** 2021-07-08

**Authors:** Claudio Conforti, Arianna Dri, Enzo Errichetti, Enrico Zelin, Iris Zalaudek, Nicola Di Meo

**Affiliations:** 1 Dermatology Clinic, Maggiore Hospital, University of Trieste, Piazza Ospitale 1, Trieste, 34125, Italy; 2 Dermatology Clinic, Santa Maria della Misericordia Hospital, Udine, 33100, Italy

**Keywords:** Dermoscopy, granuloma, foreign bodies, diagnosis, dermatology

## Introduction

Foreign Body Cutaneous Granuloma (FBCG) is a form of chronic inflammatory response of the body to various agents. From a histological perspective, it consists of a necrotic center surrounded by macrophages, epithelioid cells, and fibrous tissue. It clinically presents as a solitary infiltrated pink-reddish nodule.

## Cases Presentation

### Case I

**Figure 1:**
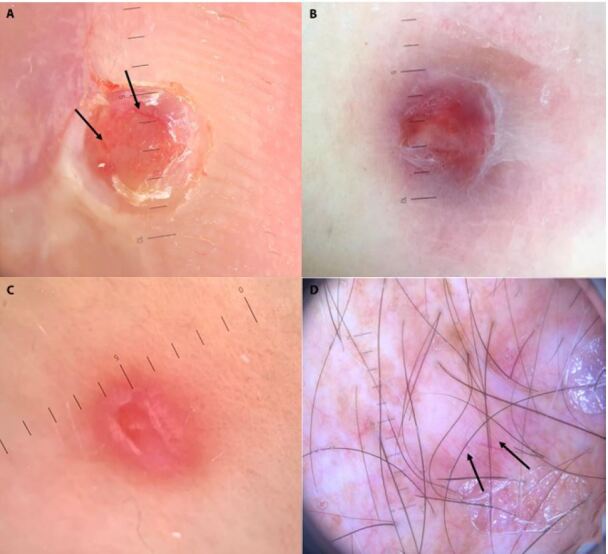
Cases’ dermoscopic examination. (A) Dermoscopic examination (DermLite 3Gen, ×10) of FBCG developed around a suture stitch and clinically configuring as a pink-reddish nodule located at the periphery of a skin graft for a previous excision of a melanoma. It configures as a structureless homogeneous milky-red area, with foci of vessels (arrows) and peripheral scales (case I). (B) Dermoscopic examination (DermLite 3Gen, ×10) of the FBCG triggered by a suture stitch, clinically presenting as a nodule on the scar of a previous cholecystectomy. It displays a homogenous bright red roundish central area, surrounded by a scaly collarette and a violaceous halo (case II). (C) Dermoscopic evaluation (DermLite 3Gen, ×10) of the FBCG including the torn of a rose, which clinically configured as a pinkish painful nodule on the wrist of the women. It is characterized by a structureless homogeneous reddish background and a central umbilication surrounded by scales (case III). (D) Dermoscopic examination (DermLite 3Gen, ×10) of the irritated papule on the leg of the young man (case IV), showing a central scar-like white area surrounded by a concentric structureless pinkish ring with linear vessels (arrows).

A 62-year-old woman came in consultation for a pink-reddish 5 mm nodule located on the sole of the right foot, at the periphery of a skin graft for an excision of a melanoma (Breslow 1.35 mm) performed 3 months earlier. Dermoscopy showed a structureless homogeneous milky-red area with vessels, a clinical diagnosis of loco-regional melanoma metastasis was hypothesized (Figure 1A).

### Case II

A 70-year-old man presented with a newly arisen red painless nodule about 5 mm in diameter located in right hypochondrium, close to a surgical scar. The patient underwent cholecystectomy 20 years ago. Dermoscopic examination highlighted a homogenous bright red, roundish central area, surrounded by a scaly collarette and a violaceous halo (Figure 1B).

### Case III

A 57-year-old woman sought consultation for a 4 mm pinkish painful nodule on the dorsal region of her left wrist. The nodule appeared 6 months ago, following a gardening session. Dermoscopy showed a structureless homogeneous reddish background and a central umbilication, surrounded by fine whitish scales (Figure 1C).

### Case IV

A 30-year-old man presented with a newly-onset, irritated, itching papule on the medial surface of left leg. He denied surgical procedures or traumas. Upon dermoscopy the lesion showed a central whitish scar-like area surrounded by a structureless pinkish ring with linear vessels (Figure 1D).

Excision and subsequent histological examination of the lesions led to FBCG diagnosis including a suture stitch (case I and II), the thorn of a rose (case III) and an ingrowing hair (case IV) respectively.

## Conclusions

To the best of our knowledge, very few reports in the literature illustrated the dermoscopic FBCG pattern, so far. Our case series highlights FBCG’s heterogeneous presentation, since it revealed reddish or bluish structureless areas, rainbow pattern, polymorphic vessels, scar-like areas, erosions, and scales.

FBCG differential diagnosis basically encompasses all entities presenting as solitary pink-reddish nodules. The appearance of new lesions on a surgical scar deriving from a previous excision should immediately raise suspicion of local recurrences and a biopsy should be performed. Similarly, a biopsy procedure must be performed if cutaneous lymphomas, Kaposi’s sarcoma, Merkel cell carcinoma, or amelanotic melanoma are suspected. Conversely, dermoscopic evaluation is usually specific enough to make diagnosis of spinal and basal cell carcinoma, dermal nevus, angioma, and dermatofibroma.

In conclusion, dermatologists should include FBCG in the differential diagnosis of pink-reddish elevated lesions in body sites that are compatible with their presence. FBCG lacks pathognomonic dermoscopic features. Still, dermoscopy represents a useful tool that can help clinicians when making an exclusion diagnosis, by ruling out conditions characterized by more specific patterns. Nevertheless, it is crucial to manage uncertain nodular lesions with excision and follow-up, these should never be considered as an option when dealing with fast growing or nodular lesions [1,2].
